# Functional and Quality of Life Outcomes in Heart Failure Patients at a Specialized Heart Failure Clinic in Dhaka, Bangladesh

**DOI:** 10.7759/cureus.85325

**Published:** 2025-06-04

**Authors:** N. A. M. Momenuzzaman, Mohammad Ashiqul Haque, Samsun Nahar, Anika Shama, Hafsa Habibi, Md. Gulam Mostafa

**Affiliations:** 1 Department of Cardiology, United Hospital Limited, Dhaka, BGD

**Keywords:** bangladesh, cardiac, cardiac output, hear failure, heart disease

## Abstract

Background: Managing heart failure patients in resource-limited settings presents significant challenges, with limited data on quality of life and functional outcomes. This study investigated these outcomes in patients attending a heart failure clinic (HFC) in Dhaka, Bangladesh.

Methods: Data were analyzed from 454 patients registered between January 2016 and December 2021 at the HFC of the United Hospital Limited, Dhaka, Bangladesh, who had baseline and follow-up data, including left ventricular ejection fraction (LVEF). A multivariable logistic regression model using generalized estimating equations identified predictors of ≥5% LVEF improvement.

Results: Patients averaged 58.4 ± 11.4 years, with 80.4% being male. Median baseline LVEF was 35% (IQR: 30-40). At baseline, 77.3% had heart failure with reduced ejection fraction (HFrEF), 10.1% had heart failure with mid-range ejection fraction (HFmrEF), and 12.6% had heart failure with preserved ejection fraction (HFpEF). LVEF improved in 39.6% of patients, while quality of life improved in 16.1%. Significant predictors of ≥5% LVEF improvement included age (adjusted odds ratio (aOR): 0.97; 95% CI: 0.95-0.99), female sex (aOR: 2.13; 95% CI: 1.21-3.73), history of acute kidney injury (aOR: 0.12; 95% CI: 0.02-0.81), and baseline HFrEF (aOR: 2.90; 95% CI: 1.34-6.26).

Conclusion: These findings underscore the need for tailored heart failure management approaches in resource-limited settings that consider patient-specific factors alongside dedicated interventions to improve quality of life.

## Introduction

Heart failure is one of the most important clinical syndromes in terms of morbidity, mortality, and costs. Globally, more than 64 million people are estimated to suffer from heart failure [1]. In Bangladesh, a meta-analysis based mostly on self-reported surveys found that approximately five out of 100 people in Bangladesh suffer from cardiovascular diseases [2]; a high proportion of these patients have heart failure. One study revealed that nearly 14% of patients admitted to a tertiary care cardiac hospital within 1.5 years were due to heart failure [3]. With increasing life expectancy and an increasing number of elderly people, the burden of heart failure may not decrease in the near future. However, appropriate treatment and proper follow-up could reduce the morbidity and mortality risk associated with heart failure.

Many pharmacological agents are now available for the treatment of heart failure and could benefit patients by preventing cardiac remodeling and increasing survival [4-6]. However, these benefits could only be facilitated through the establishment of heart failure clinics (HFCs), well-organized management programs, and large-scale prospective registries of heart failure patients [7-9]. Because follow-up assessment, dose adjustment, and drug compliance are important for treatments to work, studies have shown that HFCs can reduce the duration of hospitalization and hospital readmissions [10].

Despite significant economic progress, infrastructural development, and notable achievements in the healthcare delivery system [11], a substantial disparity persists between rural and urban health systems in the country. Consequently, healthcare-seeking behavior is not uniform nationwide, with initial referrals for emergency health issues ranging from unqualified health professionals (e.g., drug vendors, kabiraj (traditional healer), village doctors, etc.) to qualified physicians (e.g., private clinics, public hospitals, etc.) [12-14]. Primary care centers across the country are inadequately equipped to manage heart failure patients, necessitating their transfer to tertiary care centers or specialized cardiac care centers, including HFCs. Given the chronic and debilitating nature of heart failure, substantial attention and balanced management, including lifestyle modifications, are needed. However, due to the overwhelming patient load at tertiary care centers, only a small proportion of patients receive optimal care and appropriate counseling and advice beyond medication, significantly impacting their outcomes and quality of life [14,15]. Recognizing this gap, several tertiary care centers have established specialized HFCs aimed at providing standard care, proper counseling, lifestyle advice, and objective measurements of patient outcomes [16]. These clinics are designed to address the shortcomings in the current healthcare system and improve the management and quality of life of patients with heart failure.

The majority of the studies conducted on medications for heart failure were randomized controlled trials (RCTs) showing the benefits of treatment for both heart failure with reduced ejection fraction (HFrEF) and heart failure with preserved ejection fraction (HFpEF) under tightly controlled experimental conditions [17,18]. However, HFCs and hospital-based prospective registries allow monitoring of the long-term benefits of evidence-based treatments in real-world settings [7]. In addition, factors associated with improvements in heart failure could be studied from these registries. Bangladesh lacked a specialized clinic for heart failure patients until the last decade when several follow-up clinics were established. Some of these clinics have well-maintained registries of heart failure patients with baseline and follow-up data. However, few studies have explored the long-term benefits of heart failure management through HFCs in the context of low-resource settings in the country. In this study, we aimed to explore the pattern of clinical improvement in heart failure, identify associated factors, and assess changes in the quality of life and functional outcomes of heart failure patients attending a HFC in Bangladesh.

## Materials and methods

Study setting, participants, and duration

This retrospective observational study was carried out using data from the patient registry of the HFC of the United Hospital, Dhaka Limited, Bangladesh. Heart failure patients who were registered in the HFC between January 2016 and December 2021 were considered for inclusion. Those who had their baseline characteristics recorded, had been prescribed at least one medication for heart failure, and had data for at least one follow-up visit were included. Patients without baseline left ventricular ejection fraction (LVEF) data were excluded. A total of 773 patient records were screened for inclusion. Finally, data from a total of 454 patients were used for analysis.

Patient characteristics

Among the patient characteristics recorded in the registry, the following were considered for analysis based on clinical judgment and a review of previously published works [7,19]. Age and sex information were retrieved from demographic information. The etiology of heart failure was determined from the patients’ final diagnosis records. Patients with a history of coronary artery disease (CAD), angina, or myocardial infarction (MI) were deemed to have ischemic etiology of heart failure. Otherwise, they were considered to have a nonischemic cause of heart failure. Treatment history included a past history of coronary artery bypass grafting (CABG) and prescription of angiotensin receptor blocker (ARB), angiotensin-converting enzyme inhibitor (ACEI), evidence-based β-blockers, ARB neprilysin inhibitor combination (ARNI), and aldosterone receptor antagonist prior to the registry and at the registry in HFC. Comorbidity data included diagnoses of hypertension, diabetes, dyslipidemia, bronchial asthma, chronic obstructive pulmonary disease, chronic kidney disease, nonalcoholic fatty liver disease, and hypothyroidism at registration. An angiographically confirmed diagnosis of CAD, New York Heart Association classification of heart failure [20], LVEF, and Minnesota Living with Heart Failure Questionnaire (MLHFQ) score [21] were considered for clinical information. At HFC, each patient underwent an echocardiographic assessment at entry. When an echocardiographic test result was available during the month prior to enrollment in the follow-up clinic, the data from that patient were recorded. Patients are usually advised to follow up one month after the first visit, then three months after the second and third visits, and at six-month intervals thereafter. However, the follow-up time varies based on patients’ preferences and needs in the real world. In this study, for a patient with multiple follow-up visits, the last visit was taken for analysis. The duration between baseline and the last follow-up visit ranged between 1 and 14 months.

Outcome assessment

Patient outcomes were assessed by the change in LVEF assessed through echocardiography (Philips EPIQ CV, Philips Ultrasound, Inc., USA) from baseline, the increase in the walking distance evaluated by the six-minute walk distance (6MWD) test, and the addition or reduction of drugs advised for patient management. In addition, improvement in overall quality of life was also assessed by a self-reported question with four ranked categories: poor (0), medium (1), fair (2), and good (3), indicating that their condition improved versus stable versus deteriorated. Patients were asked to give a score between 0 and 3, with a higher score implying a higher quality of life. Hence, for the assessment of heart failure patients over time, follow-up data were compared with the baseline data reported at index admission.

Operational definition

Patients were categorized into three groups based on changes in LVEF from baseline to follow-up: six-minute walking test, perceived quality of life, and required types of heart failure management drugs. Based on the LVEF, patients were considered deteriorated, stable, or improved if the LVEF decreased ≥5%, remained below a ±5% change, or increased ≥5%, respectively. This cut-off point was chosen based on the lower improvement reported by DeVore and colleagues [7]. On the six-minute walking test, deterioration, stability, and improvement were defined as any reduction in walking distance from baseline, no change, and any increase in walking distance, respectively. Any improvement in perceived quality of life from baseline to follow-up was considered improvement, no change was defined as stable, and any reduction was deemed deterioration. Regarding the types of drugs used, the four pillars of heart failure (14), namely, ARNIs, aldosterone antagonists, β-blockers, and sodium-glucose cotransporter-2 inhibitors (SGLT2is), are usually given starting with one and escalating to increasing types and dosages based on patient requirements in the HFC. Patients requiring increments in the types of drugs used to control heart failure at follow-up were considered to have increased heart failure drug types, those maintained on the same drugs all throughout were considered to have unchanged drug types, and those who underwent a reduction in drug types were considered to have decreased drug types.

Patient and public involvement

Patients did not partake in the study design, developing outcome measures, or interpreting the results, nor did they contribute to the drafting or revision of the manuscript. Nonetheless, they were encouraged to assess the study questionnaire and offer constructive feedback.

Ethical approval and consent of the patient

As the study was conducted based on the secondary data recorded in the database of the HFC at the United Hospital Limited, the data were anonymized before retrieval, no active intervention was involved, and formal ethical approval was waived. Ethics approval was waived by the Public Health Foundation, Bangladesh Ethical Review Committee (PHFBD-ERC) (approval number: PHF-NG-1012). However, administrative approval was obtained before data retrieval and publication (No: UH/FEB/AO/02214; dated: February 11, 2023). All patients signed an informed consent form during registration in the HFC, permitting their data to be used in research. All procedures complied with the latest version (2013) of the Declaration of Helsinki.

Statistical analysis

We calculated changes in the ejection fraction by subtracting the baseline value from the last follow-up value. Then, these absolute changes were divided into four quartiles: the worst quartile (LVEF change: -23 to -4%), the second quartile (>-4 to 0%), the third quartile (>0 to 5%), and the best quartile (>5 to 30%). Patient baseline characteristics are presented stratified by LVEF change quartiles. Patients were also stratified according to percent change in LVEF from baseline into five categories: ≥5% reduction, unchanged, ≥5% increase, ≥5% to <10% increase, >10% to <20% increase, and >20% increase. Categorical variables are expressed as frequencies (percentages), and continuous variables are presented as the mean ± standard deviation (SD) or median (interquartile range (IQR)) wherever appropriate. Missing data was not imputed. We adopted an available case analysis approach for descriptive statistics and a complete case analysis approach for analytic statistics. The normality of the data was checked using visual analysis of the histogram with a normal curve and q-q plots. Bivariate analysis was carried out using the chi-square test and Fisher’s exact test for categorical variables and one-way analysis of variance (ANOVA) and the Kruskal-Wallis test for normal and skewed data, respectively. For paired comparisons between baseline and follow-up, the McNemar test was used for categorical variables, and the paired samples t-test was used for continuous variables.

We constructed a multivariable logistic regression model using the generalized estimating equation (GEE) method to explore factors associated with improvement in ejection fraction over time, adjusting for intrasubject variations between baseline and follow-up observations. A 5% increase from baseline was considered the response variable for the regression model. The following variables were considered factors for exploration: age, sex, presence of CAD, ischemic cardiomyopathy, dilated cardiomyopathy, New York Heart Association (NYHA) classification of heart failure, classification of heart failure based on baseline ejection fraction (HFpEF: LVEF ≥50%, HFmrEF: LVEF >40 to <50%, and HFrEF: LVEF ≤40%) [22], prior CABG, presence of comorbidities, escalation of ARB/ACEI, evidence-based β-blockers, ARNI, and aldosterone antagonists. We used the binomial distribution and logit link function in the GEE model. Rather than excluding insignificant variables found in the bivariate analysis, we kept all of the variables in the final model to account for the inherent interactions. However, significant variables in the final model were highlighted in the results. A p-value of less than 0.05 was considered significant. All the statistical analyses were carried out using the statistical software IBM SPSS Statistics for Windows, Version 26 (Released 2019; IBM Corp., Armonk, New York, USA). Graphs were built using SPSS and Microsoft Excel Version 2016 (Microsoft, Redmond, WA, USA).

## Results

Baseline characteristics of the patient with heart failure

The patients had a mean age of 58.4 ± 11.4 years (±SD), and the majority were male (80.4%). Most patients had NYHA class II heart failure (54.7%), followed by class III heart failure (33.8%) and class I heart failure (11.7%) at baseline. The mean and median baseline LVEF were 37.4 ± 9.7% and 35 (IQR: 30-40%), respectively. Based on the baseline LVEF, 77.3% of the patients had HFrEF, 10.1% had HFmrEF, and 12.6% had HFpEF. Patients were categorized into four quartiles based on the absolute change in ejection fraction from baseline to follow-up. The worst quartile, second quartile, third quartile, and best quartile constituted 26%, 30.4%, 23.8%, and 19.8% of patients, respectively. Table 1 describes the baseline characteristics of the patients stratified by LVEF change categories. Patients in the third and best quartiles had a significantly lower average age than those in the worst quartile (p = 0.004). A graded increase in the proportion of female patients was noted starting from the worst quartile to the best quartile (p = 0.006). The best quartile of LVEF change had a significantly greater proportion of HFrEF patients than did the worst quartile, which had a greater proportion of HFpEF patients (p < 0.001). However, the NYHA category of heart failure did not show any significant association with ejection fraction changes. The MLHFQ score was available for only 57 people. The median score was 11 (IQR: 5.5-21.5), which was not associated with changes in ejection fraction. Among all the patients, 76.2% had CAD, 37.4% had ischemic cardiomyopathy, 10.8% had DCM, and 14.2% had a history of CABG. Compared to patients in the worst and second quartiles, a significantly greater proportion of patients in the third quartile and best quartile had dilated cardiomyopathy (p = 0.039). Of all patients, 2.9% had a history of acute kidney injury, which showed a nearly significant association with LVEF change quartiles, with the worst quartiles having a greater number of patients (p = 0.050). The majority of the patients had two comorbidities (35.0%), followed by 30.4% with a single comorbidity, 18.7% with three or more comorbidities, and 15.9% with no comorbidities. The presence of comorbidities did not show any significant association with LVEF changes. ACEIs/ARBs, ARNIs, evidence-based β blockers, and aldosterone antagonists were newly started or had their dose increased at baseline in 56.8%, 60.8%, 52.9%, and 11.0% of patients, respectively. None of the medications showed any significant association with LVEF changes over time among the participants in this study.

**Table 1:** Baseline characteristics of the patients categorized by changes in left ventricular ejection fraction The data are expressed as the mean ± SD, median (IQR), and n (%) where appropriate. The percentage was calculated across columns. The p-value was determined by the chi-square test, Fisher’s exact test, one-way analysis of variance (ANOVA), and the Kruskal-Wallis test where appropriate. The test statistic is not applicable for Fisher’s Exact test (-); other test statistics were χ^2^ for the chi-square test, F for ANOVA, and H for the Kruskal-Wallis test. *Post hoc analysis using Bonferroni adjustments: p < 0.05 compared to the worst quartile **Comorbidities considered: hypertension, diabetes, dyslipidemia, bronchial asthma, chronic obstructive pulmonary disease, nonalcoholic fatty liver disease, and hypothyroidism LVEF: left ventricular ejection fraction; NYHA: New York Heart Association; HFpEF: heart failure with preserved ejection fraction; HFmrEF: heart failure with mid-range ejection fraction; HFrEF: heart failure with reduced ejection fraction; MLHFQ: Minnesota Living with Heart Failure Questionnaire; CABG: coronary artery bypass grafting; AKI: acute kidney injury; ACEI: angiotensin-converting enzyme inhibitor; ARB: angiotensin II receptor blocker; ARNI: angiotensin receptor–neprilysin inhibitor

Variable			Total	Changes in left ventricular ejection fraction				Test statistic	p-value
				Worst quartile, n = 111	Second quartile, n = 127	Third quartile, n = 103	Best quartile, n = 83		
Age (years)			58.4 ± 11.38	59.16 ± 10.97	60.77 ± 12.12	56.85 ± 10.29*	55.86 ± 11.13*	F = 4.54	0.004
Sex									
Male			341 (80.4)	99 (89.2)	105 (82.7)	79 (76.7)	58 (69.9)	χ^2^ = 12.46	0.006
Female			83 (19.6)	12 (10.8)	22 (17.3)	24 (23.3)	25 (30.1)		
NYHA class									
I			53 (11.7)	20 (17.1)	14 (10.1)	12 (11.1)	7 (7.8)	χ^2^ = 11.65	0.070
II			247 (54.7)	57 (48.7)	81 (58.7)	66 (61.1)	43 (47.8)		
III			153 (33.8)	40 (34.2)	43 (31.2)	30 (27.8)	40 (44.4)		
Heart failure type based on baseline LVEF									
HFpEF (LVEF: ≥50%)			57 (12.6)	26 (22.0)	19 (13.8)	10 (9.3)	2 (2.2)	χ^2^ = 43.10	<0.001
HFmrEF (LVEF: >40 to <50%)			46 (10.1)	23 (19.5)	6 (4.3)	12 (11.1)	5 (5.6)		
HFrEF (LVEF: ≤40%)			351 (77.3)	69 (58.5)	113 (81.9)	86 (79.6)	83 (92.2)		
Minnesota Living with Heart Failure Questionnaire (MLHFQ) score									
MLHFQ (n = 57)			11 (5.5-21.5)	9 (7-28)	11 (4.7-22.0)	7.5 (3.5-14.0)	15 (7.5-21.5)	H = 2.27	0.519
Medical history									
Coronary artery disease			346 (76.2)	89 (75.4)	99 (71.7)	90 (83.3)	68 (75.6)	χ^2^ = 4.61	0.203
Ischemic cardiomyopathy			170 (37.4)	54 (45.8)	45 (32.6)	43 (39.8)	28 (31.1)	χ^2^ = 6.68	0.083
Dilated cardiomyopathy			49 (10.8)	10 (8.5)	9 (6.5)	14 (13.0)	16 (17.8)	χ^2^ = 8.37	0.039
Prior CABG			64 (14.2)	24 (20.3)	16 (11.8)	15 (13.9)	9 (10.0)	χ^2^ = 5.63	0.131
Past history of AKI			13 (2.9)	5 (4.2)	7 (5.1)	1 (0.9)	0 (0.0)	-	0.050
Comorbidities**									
None			72 (15.9)	21 (17.8)	19 (13.8)	20 (18.5)	12 (13.3)	χ^2^ = 12.46	0.507
Single			138 (30.4)	40 (33.9)	36 (26.1)	31 (28.7)	31 (34.4)		
Double			159 (35.0)	34 (28.8)	60 (43.5)	36 (33.3)	29 (32.2)		
Three or more			85 (18.7)	23 (19.5)	23 (16.7)	21 (19.4)	18 (20.0)		
Medication									
ACEI/ARB									
None			76 (16.7)	24 (20.3)	27 (19.6)	15 (13.9)	10 (11.1)	χ^2^ = 6.0	0.431
New or dose increase			258 (56.8)	62 (52.5)	80 (58.0)	64 (59.3)	52 (57.8)		
Chronic use			120 (26.4)	32 (27.1)	31 (22.5)	29 (26.9)	28 (31.1)		
ARNI									
None			178 (39.2)	47 (39.8)	58 (42.0)	41 (38.0)	32 (35.6)	χ^2^ = 1.05	0.788
New			276 (60.8)	71 (60.2)	80 (58.0)	67 (62.0)	58 (64.4)		
Evidence-based β-blocker									
None			210 (46.3)	57 (48.3)	65 (47.1)	45 (41.7)	43 (47.8)	-	0.828
New or dose increase			240 (52.9)	60 (50.8)	71 (51.4)	63 (58.3)	46 (51.1)		
Chronic use			4 (0.9)	1 (0.8)	2 (1.4)	0	1 (1.1)		
Aldosterone antagonist									
None			65 (14.3)	25 (21.2)	16 (11.6)	15 (13.9)	9 (10.0)	χ^2^ = 7.87	0.248
New or dose increase			43 (11.0)	13 (11.0)	12 (8.7)	9 (8.3)	9 (10.0)		
Chronic use			346 (76.2)	80 (67.8)	110 (79.7)	84 (77.8)	72 (80.0)		

Improvement in LVEF over time

**Figure 1:**
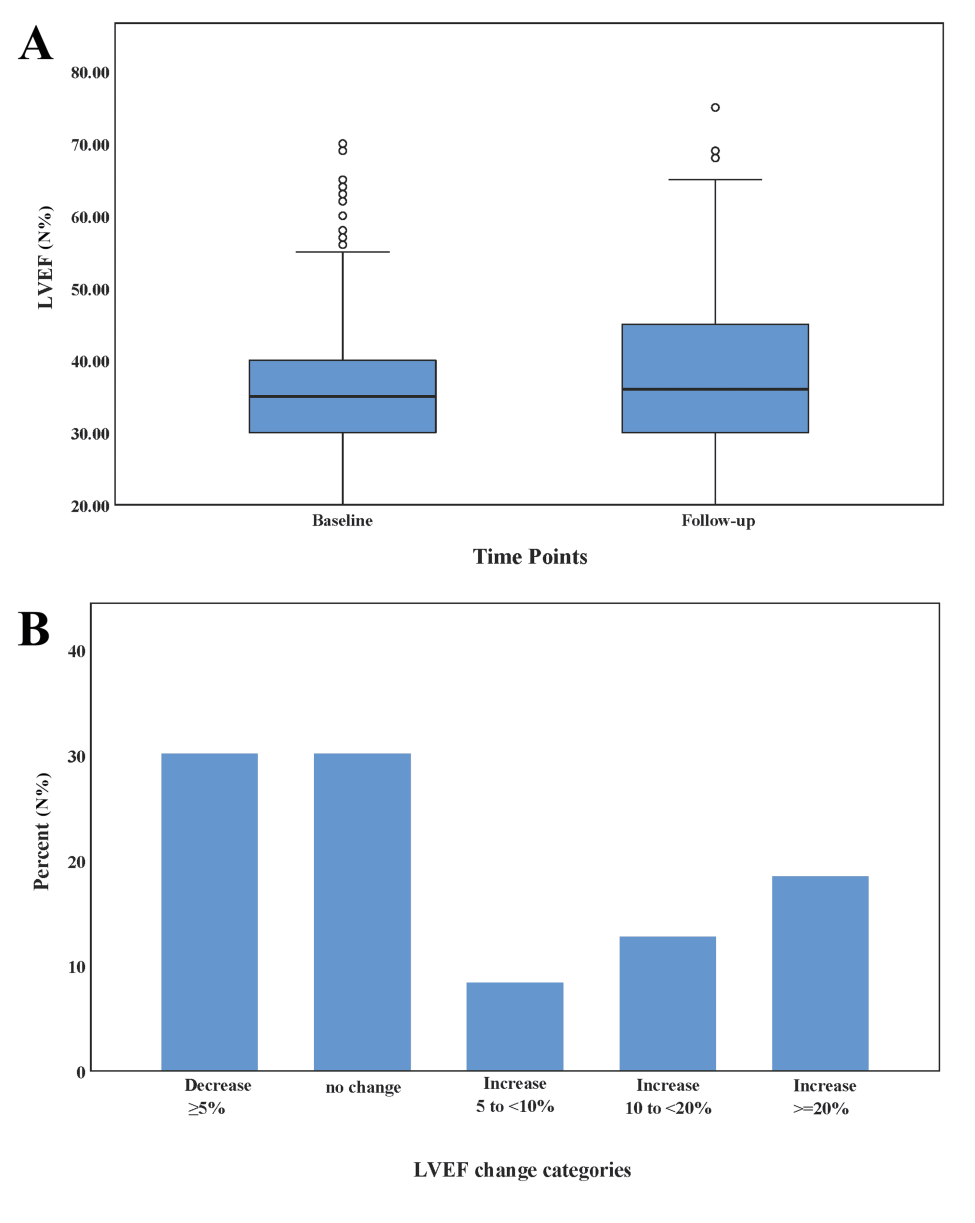
The boxplots show the median LVEF of patients with four quartiles and outliers (small circles) at baseline and follow-up (A). The bar chart shows the distribution of patients based on changes in LVEF from baseline to follow-up LVEF: left ventricular ejection fraction

**Figure 2:**
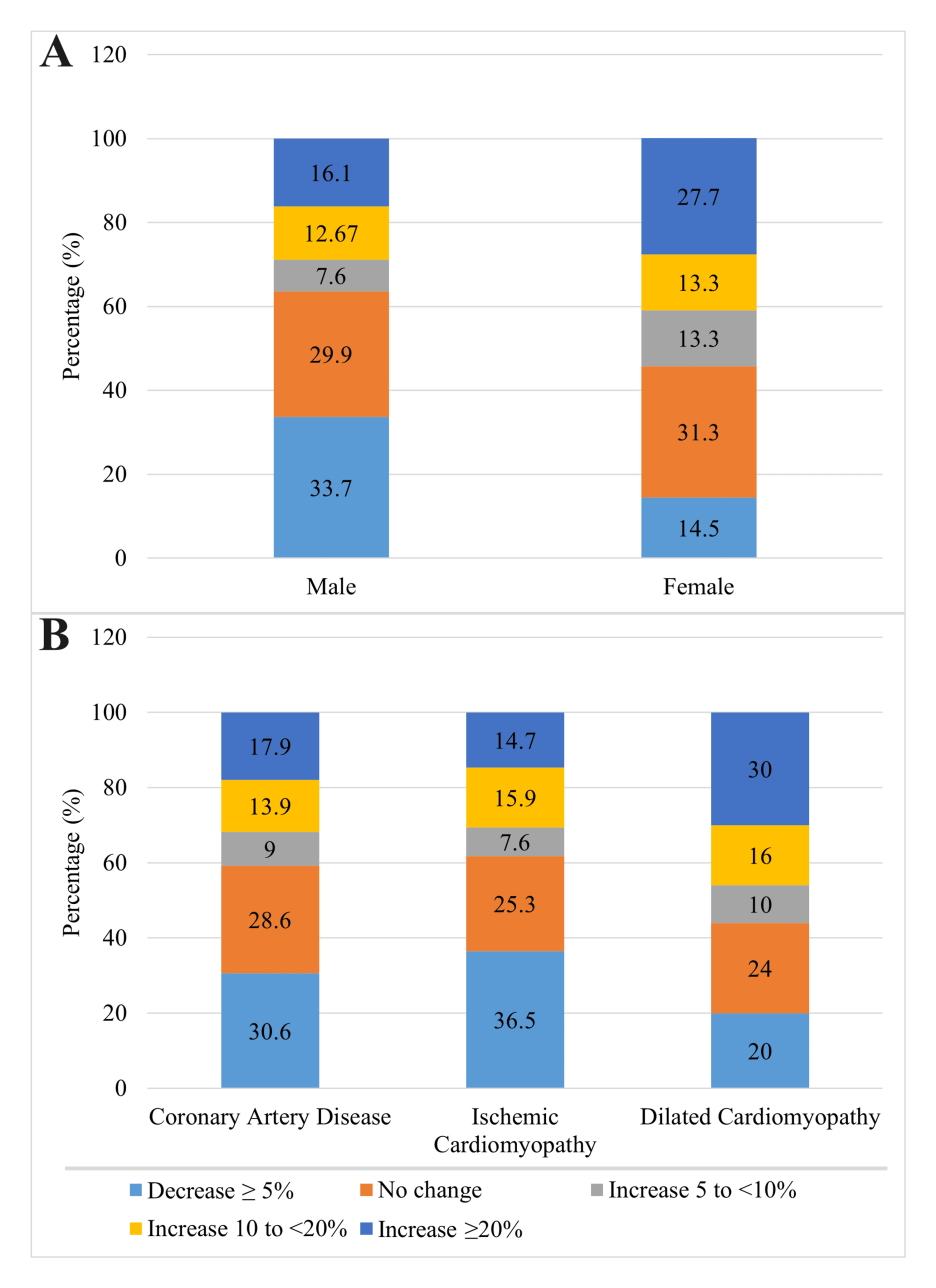
Bar chart showing distribution of patents based on changes in LVEF from baseline to follow-up stratified across sex (A) and predominant etiologies (B) LVEF: left ventricular ejection fraction

**Figure 3:**
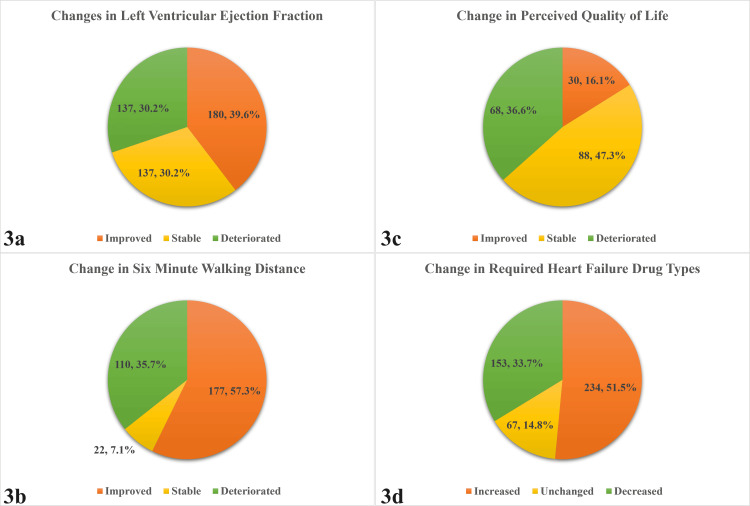
Pie charts showing changes among patients from baseline to follow-up based on LVEF (n = 454) (a), six-minute distance walking test (n = 312) (b), perceived quality of life (n = 186) (c), and required heart failure medication type (n = 545) (d). Data presented as n, % within each group LVEF: left ventricular ejection fraction

The average baseline and follow-up LVEFs were 37.40 ± 9.67% (±SD) and 38.46 ± 9.83% (±SD), respectively. Overall, 30.2% had a ≥5% decrease in LVEF, 30.2% had unchanged LVEF, 8.4% had a 5% to <10% increase in LVEF, 12.8% had a 10% to <20% increase in LVEF, and 18.5% had a ≥20% increase in LVEF from baseline to follow-up (Figure 1). The improvement was higher among female than male patients and among patients who had dilated cardiomyopathy (Figure 2). In summary, more than one-third of the patients with heart failure (39.6%) improved at least ≥5% of their LVEF over the period of time compared with the baseline LVEF (Figure 3a), and the number of HFrEF patients decreased from 77.3% at baseline to 69.2% during follow-up (p < 0.001) (Table 2).

**Table 2:** Changes in outcome measures between baseline and follow-up The data are expressed as the mean ± SD and n (%) where appropriate. The percentage was calculated across columns. The p-value was determined by the McNemar test and paired samples t-test where appropriate. Chi-square values were presented for the McNemar test and t-statistics for the paired samples t-test. *Heart failure drug types considered: ARNI, β-blockers, aldosterone receptor antagonists, and SGLT2i LVEF: left ventricular ejection fraction; HFrEF: heart failure with reduced ejection fraction; HFmrEF: heart failure with mid-range ejection fraction; HFpEF: heart failure with preserved ejection fraction; 6MWD: six-minute walk distance

Outcome variable	Baseline value	Follow-up value	Test statistic	p-value
Heart failure type based on LVEF				
HFrEF	351 (77.3)	314 (69.2)	χ^2 ^= 13.93	0.003
HFmrEF	46 (10.1)	73 (16.1)		
HFpEF	57 (12.6)	67 (14.8)		
Six-minute walking distance (feet)	1176.75 ± 334.19	1295.82 ± 555.46	t = 4.82	<0.001
Perceived quality of life				
Good	15 (8.1)	18 (9.7)	χ^2 ^= 68.45	<0.001
Fair	124 (66.7)	71 (38.2)		
Medium	33 (17.7)	72 (38.7)		
Poor	14 (7.5)	25 (13.4)		
Types of heart failure drugs prescribed*				
Four	34 (7.5)	121 (26.7)	χ^2 ^= 147.23	<0.001
Three	131 (28.9)	143 (31.5)		
Two	195 (43.0)	143 (31.5)		
One	85 (18.7)	44 (9.7)		
None	9 (2.0)	3 (0.7)		

Improvement in the 6MWD among patients with heart failure

A total of 56.7% of patients improved, and 7.9% had a stable 6MWD (Figure 3b). The average 6MWD at baseline was 1176.75 ± 334.19 ft, and at follow-up, it was 1295.82 ± 555.46 ft (p < 0.001) (Table 2).

Perceived quality of life in patients with heart failure

A total of 16.1% had improved, and 47.3% had stable perceptions of quality of life (Figure 3c). At baseline, 8.1%, 66.7%, 17.7%, and 7.5% had good, fair, medium, and poor perceptions about their quality of life, respectively. At follow-up, the proportions changed to 9.7%, 38.2%, 38.7%, and 13.4%, respectively (p < 0.001) (Table 2).

Changes in drug regimens or dose adjustment requirements

A total of 14.8% of patients required decreased doses, and 33.7% required unchanged types of drugs for heart failure control (Figure 3d). At baseline, four (7.5%), three (28.9%), two (43.0%), one (18.7%), and none (2.0%) types of drugs were used. At follow-up, the proportions changed to 26.7%, 31.5%, 31.5%, 9.7%, and 0.7% for four, three, two, one, and no types of heart failure drugs, respectively (p < 0.001) (Table 2).

Table 3 shows the results of the multivariable logistic regression model using GEEs to explore factors associated with a ≥5% improvement in LVEF from baseline to follow-up among patients. The model revealed that a one-year increase in age was associated with a 3% lower chance of having an improvement in LVEF (adjusted odds ratio (aOR): 0.97; 95% CI: 0.95-0.99). Female patients were 2.13 times more likely (95% CI: 1.21-3.73) to achieve an increase of ≥5% in LVEF over time. Those with a history of AKI were 88% less likely (aOR: 0.12; 95% CI: 0.02-0.81) to have an improvement in LVEF from baseline to follow-up. Patients with a baseline HFrEF (LVEF ≤ 40%) had significantly greater odds of having an LVEF increase (aOR: 2.90; 95% CI: 1.34-6.26) at follow-up than those with HFpEF at baseline (LVEF ≥ 50%).

**Table 3:** Multivariate logistic regression model using generalized estimating equations exploring factors associated with improvement* in left ventricular ejection fraction * ≥5% improvement **Comorbidities considered: hypertension, diabetes, dyslipidemia, bronchial asthma, chronic obstructive pulmonary disease, chronic kidney disease, nonalcoholic fatty liver disease, and hypothyroidism OR: odds ratio; CI: confidence interval; AKI: acute kidney injury; HFrEF: heart failure with reduced ejection fraction; HFpEF: heart failure with preserved ejection fraction; HFmrEF: heart failure with mid-range ejection fraction; NYHA: New York Heart Association; CABG: coronary artery bypass grafting; ARB: angiotensin II receptor blocker; ACEI: angiotensin-converting enzyme inhibitor; ARNI: angiotensin receptor–neprilysin inhibitor

Factor	Reference category	Adjusted OR (95% CI)	p-value
Age (years)		0.97 (0.95-0.99)	0.005
Sex (female)	Male	2.13 (1.21-3.73)	0.009
Past history of AKI (present)	Absent	0.12 (0.02-0.81)	0.030
Baseline HFrEF (LVEF ≤40%)	Baseline HFpEF (LVEF ≥50%)	2.90 (1.34-6.26)	0.007
Baseline HFmrEF (LVEF > 40% to <50%)	Baseline HFpEF (LVEF ≥50%)	1.30 (0.50-3.38)	0.592
NYHA class II	Class I	0.89 (0.57-1.38)	0.595
NYHA class III	Class I	0.77 (0.45-1.32)	0.335
Coronary artery disease (present)	Absent	1.58 (0.93-2.68)	0.090
Ischemic cardiomyopathy (present)	Absent	0.99 (0.63-1.56)	0.912
Dilated cardiomyopathy (present)	Absent	1.53 (0.73-3.19)	0.257
Prior CABG (yes)	No	1.00 (0.53-1.89)	0.990
Comorbidity** (number of)		1.09 (0.89-1.33)	0.434
β blocker (new or dose increase)	None or chronic use	0.96 (0.67-1.38)	0.830
ARB/ACEI (new or dose increase)	None or chronic use	1.47 (0.70-3.09)	0.306
Aldosterone antagonist (new or dose increase)	None or chronic use	1.02 (0.60-1.74)	0.944
ARNI (new)	None	0.77 (0.40-1.48)	0.434

A comparison of baseline demographic and clinical features shows a similar statistical distribution between those who had at least one follow-up (completers) and those who were lost to follow-up (non-completers) (Appendix A).

## Discussion

The key finding of our study was that nearly two-fifths (39.6%) of the patients had a ≥5% increase in LVEF over a median follow-up of 22 months (IQR: 9.5 to 35 months). The patient’s age, sex, past history of AKI, and baseline LVEF were significant predictors of improvement in ejection fraction. Other baseline characteristics, such as the NYHA classification of heart failure; the presence of CAD, ischemic cardiomyopathy, or dilated cardiomyopathy; a prior history of CABG; the presence of comorbidities; and the addition or escalation of ARBs/ACEIs, β-blockers, aldosterone antagonists, or ARNIs, were not independently associated with improvements in LVEF.

In terms of clinically meaningful improvement, the LVEF change statistics of our study are comparable to those of the Change the Management of Patients with Heart Failure (CHAMP-HF) and Registry to Improve the Use of Evidence-Based Heart Failure Therapies in the Outpatient Setting (IMPROVE-HF) studies [7,23]. The former found ≥5% LVEF improvement in 39% of patients, which is very similar to our study. The latter showed a 10% improvement in 28% of patients. Multidisciplinary HFCs have been shown to provide long-term clinical benefits in heart failure patients [7,10,24]. Hence, the establishment of HFCs and the initiation of heart failure management programs or registries based on those clinics could be considered compulsory additions to the management of heart failure.

The heart failure patients included in this study were on average in their 60s, which corresponds with the findings of Kabiruzzaman et al. [3] in Bangladesh. The incidence of heart failure increases considerably with each decade of life [25]. Hence, any HFC should expect a greater proportion of older adults and elderly patients to experience heart failure outdoors. We found that each year's increase in age is associated with a decreased chance of improvements in LVEF, which is contrary to the findings of DeVore et al. [7], who conducted a similar study and did not discover such an association. However, their study used data from HFrEF patients only, whereas we included all types of heart failure patients. The inclusion of HFpEF patients in our study might have been an important modifier of odds in our model. Increasing age is associated with lower left ventricular compliance [26] and a greater incidence of HFpEF [27].

More than 80% of patients in this study were male, which conforms to the findings of Kabiruzzaman et al. [3]. This finding is contrary to what one would expect considering the similar risk of heart failure in both sexes [28]. However, these sex differences could be related to the gender gap in health service utilization [29] and differences in risk behaviors such as smoking, which are more prevalent among males [30] in Bangladesh. Nevertheless, similar to the CHAMP-HF study by DeVore et al. [7], we found that female patients were significantly more likely to have an improvement in ejection fraction. Previous studies have shown that pharmacokinetic and pharmacodynamic differences in drug therapy can enhance improvement in LVEF in women more than in men [31], explaining the greater odds of improvement found in our exploratory study.

The baseline LVEF is an important determinant of a positive change in the ejection fraction during follow-up. We found that HFrEF patients were significantly more likely to achieve a 5% improvement in LVEF than those with HFpEF. In this context, a discussion of the effectiveness of various drugs in different types of heart failure might shed some light on the difference. In our HFC, ACEIs, ARBs, aldosterone receptor antagonists, evidence-based β-blockers, and ARNIs are the most commonly prescribed drugs that are newly added or given in an increased dose at the registry based on clinical judgment for the management of heart failure. However, Zheng et al. [18] in a meta-analysis of RCTs showed that the clinical course of HFpEF is not affected by the use of ACEIs or aldosterone receptor antagonists. Only β-blockers demonstrated the greatest reduction in all-cause and cardiovascular mortality in this group. On the other hand, another meta-analysis of RCTs by Tromp et al. [17] reported that the combination of β-blockers, ARNIs, mineralocorticoid receptor antagonists, and SGLT2is was highly beneficial for patients with HFrEF. As our study considered both HFrEF and HFpEF patients during the analysis, our findings possibly conform to these reports. However, our analysis also revealed that none of these agents were independently associated with LVEF improvement in patients. These findings are also supported by the reports from the CHAMP-HF study [7]. Likewise, a prior history of CABG was not independently associated with improvement in LVEF. The reason behind not finding any independent effect of drugs or interventions on the ejection fraction is not clear. However, patient factors, such as lack of medication compliance and irregular follow-up, and physician factors, such as inadequate dose titration and nonadherence to standard treatment guidelines, could be possible reasons for these findings and need to be further explored. Another possible reason could be the low amount of patient data that was used to build the model. Nevertheless, the fact that patients are being treated with successful improvement in ejection fraction without the prescription of digoxin in real-world settings endorses the effectiveness of the fantastic four drugs, namely, beta-blockers, ARB/ACE-I, ARNI, and mineralocorticoids [32], as well as creates an opportunity to discontinue digoxin among many heart failure patients with reduced ejection fraction. Thus, the potentially toxic effects of digoxin [33] can be avoided in many patients.

Another insight derived from our study is that any history of acute kidney injury in heart failure patients was associated with a significantly lower chance of improvement in LVEF. Patients with sudden renal function decline in heart failure (often referred to as cardiorenal syndrome (CRS)) have been shown to have a worse prognosis [34], which was reflected in our study. CRS complicates treatment by fluid overload and diuretic resistance and requires carefully planned and individualized management strategies. Effective therapeutic options for CRS are still lacking [34].

On bivariate analysis, we found that dilated cardiomyopathy patients were more likely to experience a greater improvement in ejection fraction. Ischemic cardiomyopathy patients did not show any significant association with LVEF changes. The CHAMP-HF [7] and IMPROVE-HF [23] studies noted that LVEF improvements were greater among heart failure patients with nonischemic etiology. Our findings support their findings.

The improvement in the ejection fraction itself is not the end of the story if it does not translate to improvements in the quality of life in the long run. Our study found that more than half of the patients either had a stable or improved quality of life after being managed at the HFC. The guideline-mediated therapies with the fantastic four drugs in isolation or combinations have been shown to achieve remarkable improvements in mortality and quality of life [32]. Our experience in the HFC of Bangladesh has been similar.

We noted that during this five-year period, nearly 40% of the patients enrolled in the registry did not return for a second visit. The reasons behind such a high number of patients lost should be explored. Health education activities could be added as a part of the treatment plan to reduce follow-up loss among patients. Subsidized costs or insurance-based health services could be considered if cost is a factor.

Limitations

This study has several limitations that warrant discussion. The retrospective design introduces selection bias, as patients attending our HFC likely represent a more health-conscious subset with better healthcare access. Without randomization or a control group, causality between interventions and improvements cannot be established. Measurement variability in echocardiographic assessments may have influenced LVEF results despite being performed at the same center. Our analysis lacked data on critical confounders, including medication adherence, duration of heart failure before enrollment, socioeconomic status, and health literacy. The variable follow-up time and high attrition rate (40% not returning after the initial visit) suggest follow-up bias, potentially overestimating treatment benefits. Using subjective self-reported quality of life measures rather than completed MLHFQ scores introduces social desirability bias. The relatively small sample size limited statistical power, particularly for subgroup analyses. Finally, as a single-center study at an urban tertiary hospital, our findings may not generalize to rural settings or different healthcare contexts in Bangladesh. Despite these limitations, this research provides valuable initial insights into specialized heart failure management in Bangladesh and establishes the groundwork for more rigorous future studies.

## Conclusions

We observed improvements in the LVEF, the ability to walk longer distances, and, among a subset of patients, positive changes in quality of life and reduced drug use among our patients visiting the HFC. Chronological age, sex, and baseline ejection fraction at admission were significant predictors of overall improvement in the patients. However, this finding should be read taking into consideration the observational nature of the study, which cannot establish causality but can generate hypotheses for further evaluation. The limitations described should also be kept in mind while interpreting our findings. Despite limitations, this study was one of the earliest attempts to investigate the impact of managing heart failure in HFC and associated factors in Bangladesh. The findings of this study will add to the growing body of literature on myocardial recovery. It could encourage clinicians and policymakers to engage in discussions regarding heart failure management strategies to determine an optimum guideline and solution tailored to the needs of the community.

## Appendices

Appendix A 

**Table 4:** Comparison of baseline features between patients who completed follow-up and those who did not complete LVEF: left ventricular ejection fraction

Characteristics	Follow-up		p-value
	Non-completers	Completers	
	Mean ± SD or n (%)	Mean ± SD or n (%)	
n (%)	317 (41.0)	454 (59.0)	
Age (years)	58.06	59.31	0.425
Sex			
Male	210 (78.9)	341 (80.4)	0.638
Female	56 (21.1)	83 (19.6)	
Etiologies			
Coronary artery disease	212 (66.5)	346 (76.2)	0.003
Ischemic cardiomyopathy	116 (36.4)	170 (37.4)	0.759
Dilated cardiomyopathy	36 (11.3)	49 (10.8)	0.829
Acute kidney injury	9 (2.8)	12 (2.6)	0.881
Comorbidities			
None	49 (15.5)	72 (15.9)	0.076
One	88 (27.8)	138 (30.4)	
Two	96 (30.3)	159 (35.0)	
Three or more	84 (26.5)	85 (18.7)	
LVEF (%)	37.79 ± 9.89	38.00 ± 18.92	0.833
